# Observations on Diphtheritis

**Published:** 1859-05

**Authors:** 


ART. II.—*Observations on Diphtheritis.* By W. F. WADE, B.A., M.B., T.C.D., Physician to the General Dispensary, formerly Resident-Physician to the General Hospital, Birmingham. 8vo. London: John Churchill, 1858.

*Diphtheritis: A Concise Historical and Critical Essay on the late Epidemic Pseudo-Membranous Sore-Throat of California, etc. etc.* By V. J. FOURGEAUD, M.D. 8vo., pp. 44. Sacramento, 1858.

WHAT is diphtheritis—what is the form of disease thus named in these two essays from the antipodes—the one just issued from the Sacramento press, the other from New Burlington Street, London? Do the writers treat of the same morbid affection thus showing itself in opposite hemispheres, or do they use one word to convey different meanings? Let us analyze the pages before us, and extract in brief the instruction they convey.

Mr. Wade sets out apparently with the view to establish "a foregone conclusion," which he announces clearly enough in his preface, the essential presence of albuminuria as an element of the epidemic of which he treats, and which he distinctly maintains to be identical with the diphtheria of Dr. Farr, the diphtherite of Bretonneau, the malignant ulcerous sore-throat of Fothergill, the cynanche maligna of Cullen and Johnstone, the angina suffocativa of Bard, and the gangrenous sore-throat of Chomel. It would be tedious to enumerate the other titles by which he assumes it to have been designated at other times, and by other authorities. He proposes these novel views as "a basis upon which to erect a pathological and ultimately a therapeutical structure."

The name accepted generally, as it is by our author, was originally assigned to the disease by Bretonneau, of Tours, from its most obvious characteristic—the exudation of a plastic or pellicular deposit on the mucous surface of the fauces and pharynx—after the Greek Διφθέρα, a skin membrane. Farr calls it diphtheria, an innovation against which Mr. Wade protests with some learned objections.

His description of the attack, a full and graphic one, can scarcely be abbreviated without injustice. He dwells upon the symptoms of general or constitutional disorder which mark the invasion; directs our attention to the local characteristic affection soon supervening; "the discomfort rarely amounting to pain in swallowing; the enlargement of the tonsils, generally of a dull-red color, and studded with whitish specks; the follicles may be dilated, giving rise to the impression that the tonsils are ulcerated; or the whole mucous membrane of the tonsils, soft palate, back of the pharynx, uvula, hard palate, and even portions of the tongue and cheeks may be covered by a thick tenacious film." This false membrane is naturally "white or whitish, but may acquire a dark hue from imbibition of blood, or colored drinks, or desiccation. It may be removed sometimes, so coherent is it, in large patches, by means of the forceps, and the membrane beneath, to which it is more or less adherent, will be found of a shade from dull purple to fiery red, and tumefied. The fetor of the breath, so much spoken of, is not universal; it depends, probably, sometimes at all events, upon partial decomposition of the lymph or fluids secreted by the mucous membrane." A red papular eruption, of brief duration, uncertain period of appearance, and limited extent, may attend; diarrhœa often presents itself; "the urine is generally pale, rather scanty, but to the eye normal; occasionally, however, high colored, and loaded with lithates." The glands of the neck are apt to become enlarged and painful, and may suppurate; "an acrid offensive sero-sanguinolent discharge issues from the nose, the angles of which ulcerate, the ulcers being coated sometimes with lymph, as are also the surfaces of blisters when they have been applied.

"The patient not unfrequently dies when the disease is apparently very slight, or when convalescence is well established or even seemingly almost complete. This feature of the complaint it is which especially renders it disagreeable to treat and difficult to manage." "Croupy symptoms occur finally in almost all fatal cases, whether at first they were slight or severe." "A purpuric condition is often manifested in the later stages, with hemorrhage from the nostrils, bowels, or kidneys." Regurgitation of fluids through the nose when attempts are made to swallow, and a nasal tone of voice, which belong to all periods of the attack, may persist for a long time after the patient is in other respects well. "The duration of this disease is very uncertain; its most fatal period is from the sixth to the eighth day, but the patient may linger on, and finally die several weeks from the commencement of the attack. Children are chiefly, but not exclusively, its subjects: "it is usually epidemic; spring and autumn are most favorable to its ravages."

All readers who have examined the subject, have found it difficult to draw clear lines of distinction between several forms of disease which present in common this affection of the throat: thus between scarlatina anginosa and malignant sore-throat there has always been confusion, and we have seen that our author recognizes the identity of diphtheritis with the latter. Bretonneau, while he admits the pathological relations of his diphtherite with scorbutic gangrene of the gums and croup, strenuously denies that there is any connection between it and scarlatina. The diagnostics upon which he lays special stress, however, are both uncertain: the characteristic appearance of the exudation in each; and more particularly the contrasted relation to croup which he affirms of the two affections. "Finally," is his strong phrase, "finally, there is a more important differential characteristic of the scarlatinal inflammation of the pharynx; it has not any tendency to propagate itself into the air-tubes."

Our author, while doubtless regarding diphtherite as a separate and specific disease, disposes briefly and impartially of both these assumptions of his French predecessor. "The condition of the fauces," he says, "affords no reliable distinctions between the two diseases." He then goes on to establish, by abundant authority, the coincidence of croup with scarlatina, or its supervention during the course of that exanthem, and concludes that there have happened "epidemics of malignant sore-throat undoubtedly congeneric with scarlatina," as well as "others, such as that at Tours, or the present one, in which this connection is less manifest and perhaps questionable."

Mr. Wade next sets himself to establish "the production of false membranes or fibrinous exudation by an inflamed surface, and examines the question whether the older writers were right in calling the appearance which they described, *sloughs.*" Fothergill maintained that there was real "mortification of the substance, not a crust or coat of foreign matter." Our author, regarding the fact of ulceration as unimportant in this connection, and the fetor already spoken of as "no criterion," lays principal stress on the absence of any history of destruction of the soft parts, and denudation of the palatial bones, some or all of which must have repeatedly happened in the course of such gangrene; and decides that "Fothergill and others must have mistaken exudation on the mucous membrane for sloughs of this substance."

In the chapter on Symptomatology we find the malady divided into three stages: *first,* the initial; *second,* that in which the exudation has appeared in the throat; and *third,* the terminal, indicating the conclusion, whether favorable or the "reverse."

1. The initial symptoms, varying greatly in degree, are such as "usually attend febrile disorders—chilliness, anxiety, giddiness, nausea, and vomiting." Our author sets down as important points, that the initial fever is almost invariably present; that the febrile reaction is "out of proportion to, I mean less than, the preceding depression or rigors; that it precedes the faucial exudation; and that it bears no proportion whatever to the amount of the exudation." "That it is really lymph spread upon the mucous membrane is proved, first, by microscopical examination, which shows it to consist of the fibrillar and corpuscular elements of plastic lymph in varying proportion, and by stripping it off, leaving the membrane perfect underneath." Dr. Laycock, in examining "the pellicle formed on the tongue and fauces of a case of diphtheria or diphtherite, found the sporules and mycelium of the *oidium albicans*, a parasite fungus, found also in muguet, the epidemic aphtha or diphtheria of infants in France." Dr. Laycock, as we conceive, mistaking consequence for cause, ascribes the production of this pellicle "to the action of the parasite on the enfeebled mucous surface of the mouth and fauces. It acts," he goes on to say, "like all its tribe, as an irritant, inducing increased formation of epithelial scales and effusion of mucus, exudation corpuscles, or plasma, intermingled among which are the sporules and mycelium of the microscopic fungus." Mr. Wade found in a similar case "the minute alga *Leptothrix buccalis* in great quantity, but not a trace of the *oidium*."

We pass, of necessity, over many of the details of symptoms and their progress, carefully noted and recorded by our author, and come to the point upon which turns the chief interest of his inquiries, in his own estimation. This was the discovery of the presence of albuminuria—the correctness of which observation has been since confirmed, he tells us, by many medical gentlemen whose names are referred to, "indicating a connection between diphtheritis and acute desquamative nephritis. The specific gravity of the urine varies, but it may be laid down pretty certainly that there is a diminution in the total amount of solid excretion; or, in other words, that the special functions of the kidneys are interrupted. Although the urine is often perfectly pellucid and free from deposit, yet in some instances we find what I should certainly be disposed to anticipate in all, viz., tube casts and renal epithelium. The tube casts may be of three forms: first, small waxy casts; secondly, casts of similar size, but granular, probably from commencing disintegration; and thirdly, ordinary epithelial casts. Besides these objects, and perhaps even more common than these, are small masses of irregular shape, of fibrin, not moulded in the renal tubes; epithelium also from the bladder, etc. The tube casts and the glandular epithelium often contain fat in a state of subdivision more or less minute."

The next inquiry, and an important one doubtless, is as to the value, prognostic and therapeutic, of the discovery thus alleged to be made. Albuminuria is not a fatal symptom; the quantity of albumen in the urine is generally proportional to the functional disability of the kidneys, a sign of a condition interfering with their physiological activity. "We must not overlook the possibility of the renal offices being supplemented by other organs." Mr. W. mentions two chronic cases, "one of which died at the end of five weeks, and the other did not begin to amend till nearly eight weeks after the first invasion; in both these the albuminuria was present and persistent, until a day or two before death in one which was fatal, and in the other its disappearance was the first indication of returning health."

Roche speaks of a case which lasted for eight months; another is given on the authority of Girouard, of two years continuance. In neither of these do we see any notice taken of the urine; and, indeed, it may be doubted whether such instances present any other than the mere local affection. We had under our care a lady whose throat was coated for fully six months with a thick yellow buckskin exudation; the constitutional symptoms subsided after ten or twelve days, leaving her with pharyngeal uneasiness and dysphagia, occasioned by the persistent presence and prompt reproduction when removed, of this tenacious pseudoplasm.

Our author seems disposed to ascribe to uræmic poisoning the peculiar phenomena described as presenting themselves in bad cases everywhere: Cullen's "symptoms of putrid fever," Bard's "great and sudden prostration of strength, with comatose and lethargic condition," "the lethargy" of René Moreau, and the "stupor" of Bartholinus. He refers in this connection, "without prejudging the question of the "relationship between scarlatina and diphtherite," to the gigantic "work of Dr. Copland, for information upon the presence of albuminuria in malignant scarlet fever, a complication upon which he much insists, and to which he attributes many of its phenomena."

Among the modes of death enumerated, are suffocation by the extension of the membrane into the larynx, croup, œdema of the glottis, and "the dislodging of some of the masses of lymph, which might either close up the opening of the glottis or fall into the larynx." Besides croup, the most frequent of complications in all countries, we meet occasionally with convulsions, almost invariably fatal, and diarrhœa, which is attended with great danger.

Frequent micturition is also included among the most threatening symptoms, as well by Bard as by our English author. Purpura and hemorrhages are frequent forerunners of death; but Mr. Wade affirms that he has seen no case of purpura in which the kidney affection had not previously shown itself. He closes his interesting and carefully labored essay with a promise to "endeavor to elucidate, at some future period, the pathology and treatment of this complaint."

We turn now to Dr. V. J. Fourgeaud, well known to us as an able editor, in former days, of one of our most respected western journals, and a practitioner of extensive reading and marked ability. In his "Historical and Critical Essay" we note, at the outset, two points which must strike every reader with much force: the absence of every reference to that condition of the renal excretion which the English pathologist regards as of paramount importance; and secondly, the tendency to consider the disease as primarily local, and the symptoms consequently involving the constitution, and endangering the life of the patient, as secondary results, absolutely dependent upon the throat affection, and the effects merely of its extension and interference with the functions.

The history of the epidemic of 1856, hastily but graphically sketched, leads us at once to the doctrine thus taught, and the gratifying results of the practice deduced from it.

"Among the districts suffering most, was that of Sonoma. A friend of mine had lost three of his daughters within a few days. Determined at once to hasten to the assistance of the afflicted family, I arrived too late, however, for one of the still surviving daughters, whom I found in articulo mortis, and beyond the resources of our science. I at once diagnosticated the disease to be well characterized pharyngeal diphtheritis, described by Bretonneau, and, encouraged by the wonderful success which had followed his treatment in similar epidemics, I did not hesitate to place my main reliance on local cauterization, and like him I selected muriatic acid as the agent. The result proved even more satisfactory than I could have reasonably anticipated. Of all the cases that have come under my observation from that time, *not one* has terminated fatally. Indeed, so successful has been this treatment in California, when resorted to early, that I now consider pharyngeal diphtheritis, such as it has been observed in this State, among the most manageable, and, when properly attended to, least fatal of diseases."

Lest the reader should doubt whether our two authorities are treating of the same malady, we will give a brief abstract of Dr. Fourgeaud's luminous account of the California epidemic. We have said that he recognized it at once as Bretonneau's diphtherite, ("from a Greek word, signifying membrane,") and proceeded to treat it accordingly. He reviews its history, tracing it as far back as the time of Aretæus. He admits its "great analogy with the putrid sore-throat that often accompanies malignant scarlatina," but declares that "it is not the same malady, and it is important to make the distinction. It differs essentially from it. Ulceration of the throat is very rarely observed in this disease." He states, however, that "when scarlatina and diphtherite appear at the same time in a locality, the patients affected with the first are predisposed to the latter."

He regards "membranous sore-throat as in some degree contagious. Girls are more frequently affected than boys," though this is not always remarked. "It may attack persons of all ages, but children of feeble constitution and of lymphatic temperament are most predisposed to it."

In recounting the symptoms, he dwells upon its commencement as a local affection. "The disease begins in a very insidious manner, by a little engorgement or inflammation of the soft palate, pharynx, and one of the tonsils. The attack seldom commences on both, but soon extends to both if not arrested." And again, "the disease begins with the inflammation of the mucous membrane of the soft palate, tonsils, and pharynx, terminating in the secretion of a false membrane, without ulceration or destruction of the skin," p. 8. "After the period of invasion, more or less fever is observed," etc. "There is no vomiting nor diarrhœa unless the mucous coat of the stomach and intestines becomes the seat of disease." "In favorable cases, recovery generally commences before the disease reaches the larynx, trachea, and bronchi; when the false membranes are produced on these parts the patients seldom recover."

According to Guersent and other pathologists who have made post-mortem examinations in this disease, the morbid appearances observed are not always confined to the pharynx, larynx, and trachea, but false membranes are sometimes discovered in the œsophagus, stomach, and intestines. Dr. Fourgeaud tells us, however, that "these gastro-intestinal complications are of rare occurrence," and begins his observations on the treatment with the rule that "this disease being, as we have seen, originally local, confined to the mucous membrane of the soft palate, tonsils, and pharynx, the treatment must be principally local." And again: "as I have observed before, the most important and effectual treatment is the local application of acids." "I have used successfully pure hydrochloric acid twice a day in the case of a child twelve years old, in which the tonsils, the soft palate, and a considerable portion of the pharynx were covered with the false membrane. It acted at once in arresting the further progress of the affection by circumscribing it, as it were, in the limits it occupied. From that moment the general symptoms gradually improved." Not that his treatment is always exclusively local. He tells us, on the contrary, that he has found benefit in an early emetic, and in the use of calomel and quinine.

A portion of Dr. Fourgeaud's interesting essay consists in a controversial examination of the views of some of his Californian brethren. Dr. Hardy, of Monte Christo, described a sore-throat epidemic in his locality. We have not before us Dr. Hardy's paper, and therefore will not comment upon Dr. Fourgeaud's conclusion, that the disease thus discussed "was not primitive or true diphtheritis, but one of the secondary forms of angina, such as usually accompanies scarlatina and other eruptive diseases."

In direct opposition to the views of Dr. Fourgeaud are those of Dr.

Blake, contained in an article published in the *Pacific Med. and Surg. Journal.* Dr. Blake maintains the constitutional character of pharyngeal diphtheritis, which, he says, "is as much a systemic disease as typhoid fever," and absolutely sui generis. Against this doctrine the authority of Valleix is produced, and that of Rilliet and Barthez, all of whom certainly speak of the malady as a local affection and dangerous by extension chiefly, if not exclusively, and by its mechanical interference with respiration. These latter admit the occasional assumption of a typhoid or adynamic aspect, but ascribe it, as do Bourgeois and others, to the poisonous effects of "putrefied secretions from the mucous membrane."

In "Ranking's Abstract," vol. xix. p. 192, there is a somewhat extended notice of Dr. Camp's essay "on the lately prevailing Diphtheritic Affection," the same, doubtless, described by Dr. Wade, recognized as identical with Bretonneau's, Fuller's, and Brown's, as extending itself over several of the counties of England, as well as the metropolis. It serves well to show the uncertainty of medical opinions, and the propriety of guarded expressions in speaking of disease as it appears under our own observation, that Dr. Camp considered the affection as "mainly, if not effectually, of an asthenic, adynamic type." We have seen Dr. Fourgeaud maintaining strenuously the opposite view, and supporting himself strongly by the great mass of French authorities; he quotes also from Professor Dunglison a brief, explicit statement, in separating gangrenous from diphtheritic pharyngitis, that the former is "eminently typhous, and the decided difference between gangrenous and diphtheritic pharyngitis, after all, consists in this circumstance."

Mr. Brown, of Haverford West, had no fewer than two hundred cases in 1849, of which he lost forty; in all of whom "the pharynx and larynx were simultaneously affected;" post-mortem examinations showing these parts with the tonsils, trachea, and bronchi coated with plastic, pseudo-membranous exudation. His treatment was both constitutional and topical. He administered calomel with ipecacuanha, and affirms that he "did not lose a patient in whom he succeeded in establishing ptyalism." He found early emetics also very useful. He advocated the topical application of the solution of nitrate of silver.

The therapeutic of Dr. Camp was very similar. He employed "free applications of a strong solution of nitrate of silver to the parts affected, composed of from ℈i to ʒij of the nitrate to one ounce of water, or similar applications of chlorine or hydrochloric acid; with the repeated administration of chlorate of potassa, or chlorine, or a combination of cinchona or its alkaloid salts with the mineral acids, and in the rarer cases calomel in repeated doses so as to produce ptyalism. An emetic may be occasionally given with advantage in the early stages; and when the disease has continued some time, the vital powers must be sustained with wine, stout, beef-tea, and other invigorating means."

In the *Am. Journ. Med. Sciences*, October, 1858, we have an interesting abstract of a paper by Isambert, on the Paris epidemic of 1855. He speaks of diphtheritic affections as spreading, endemic, epidemic and contagious. He regards them as of specific character, the general aspect or tendency being adynamic and typhoid. Relapses are not rare. Cutaneous counter-irritation is prohibited, because of the diphtheritic manifestations ready to show themselves, and become new centres. The general management is by emetics, mercurials, alkaline carbonates, and chlorates. Local applications of calomel, alum, hydrochloric acid, and especially nitrate of silver are recommended. The patient should be well nourished and sustained by bark, coffee, and wine.

A brief but concise history of the same epidemic, a diphtheritic angina, as it was called, which prevailed in Lima, is given us from the pen of Odriozola, a Spanish physician of that city. It showed itself first in 1855, and reappeared in 1858.

We meet with it, thus, in all climates, in almost all seasons, and under very varied contingencies. But in Lima it presented some singular features. It attacked few of the lower classes, the poor, the most exposed, assailing "in preference those who enjoyed all possible comforts." The black race, we are informed by the same writer, "has been as resistant to this angina as to the yellow fever." Children were its principal subjects, though not exclusively, for there were two adult deaths from it. "Cauterizations with pure hydrochloric acid, practiced two or three times a day, and emetics of tartarized antimony or ipecacuanha were alternately employed. The deaths were about one in five or six."

A pithy paragraph from the *Med. Times and Gaz.*, October, 1858, conveys a most unfavorable view of the prognosis and influence of treatment in this formidable malady in Paris:—

"In thirty cases of diphtheritis observed at the Hospital St. Eugenie, medicine does not seem to have been of any service. Large doses of ipecacuanha were given in vain; so also calomel and alum; chlorate of potassa was found useless. In four patients, the internal surface of the larynx was cauterized several times with nitrate of silver, by aid of M. Loiseau's instrument, but the operations were not followed by the effects noted by its inventor. In the great majority of the cases tracheotomy was had recourse to."

Having thus laid before our readers a general outline of the state of medical opinion and practice in reference to this subject, we proceed to offer the result of our studies in a brief and condensed form:—

1. It seems to us, then, that in the present condition of our knowledge, it is safer to speak of diphtheritis as a diathesis, tendency, or symptomatic manifestation of a morbid character, than as a specific or separate disease. In the fauces, we have diphtheritic pharyngitis; in the larynx, membranous croup; in the intestines, tubular diarrhœa. A diphtheritic conjunctivitis has been described; membranous pseudo-plastic exudations occur in scarlatina and angina maligna; they may appear, says Valleix, "in rubeola, sometimes; more seldom in variola, and still more rarely in typhoid fever." West tells us that he "once found the œsophagus lined by a complete tube of false membrane to within an inch of the cardiac orifice of the stomach." We saw a large mass extruded from the bowels in a case of yellow fever. The nipples of a nursing mother have become diphtherous from contact with the mouth of a diseased child. Blisters are covered with this exudation in diphtherous patients. Nay, Dr. Fourgeaud quotes a case in which "a child at the College of La Fleche, while suffering from chilblains during an epidemic of diphtheritis, had his toes affected with diphtheritic concretions."

2. This pseudo-membrane, consisting as it does of a plastic exudation, albuminous, fibrillary, mingled with inflammation corpuscles, pus globules, and, as Seitz tells us, "a species of cell, double the size of the pus globule, but otherwise similar to it," is of various degrees of organization or organizability. It exhibits various degrees of adhesion to the surface from which it is thrown out, being sometimes tenaciously connected with it, and sometimes very loose. This connection is probably vascular, and denotes its degree of vitality, or rather, so to speak, its potentiality of assuming a dependent life.

3. It is liable to become the seat of varied parasitic development, algous or fungous.

4. It is occasionally associated with contagious diseases.

5. Hence, either by such complication, or by actual contact, or transmission, it seems to be conveyed from one person to another.

6. It is usually, however, endemic or epidemic; depending on some of those obscure conditions surrounding us locally, with which we are familiar, without at all comprehending them.

7. It is often surprisingly amenable to local treatment.

8. It is as often singularly indomitable, refusing to be controlled by any method of management whatever.

9. The mercurials seem to have been most generally found serviceable of all constitutional remedies.

10. Of local remedies, the hydrochloric acid stands first in general estimation; next the nitrate of silver; then the sulphate of copper; alum and the perchloride of iron are also recommended.

11. Emetics are extensively used; partly for their local, partly for their constitutional influences.

12. The albuminuria observed by Mr. Wade is probably a part of the general tendency to albuminous exudation or exosmose. Its presence should be inquired for by all observers.

13. The phenomena of pseudo-plastic formation may be connected either with sthenic inflammation, or asthenic, adynamic constitutional conditions.

